# Effectiveness of home-based exercise in breast cancer survivors: a randomized clinical trial

**DOI:** 10.1186/s13102-023-00710-7

**Published:** 2023-08-07

**Authors:** Vahid Farajivafa, Nasim Khosravi, Nilofar Rezaee, Maryam Koosha, Shahpar Haghighat

**Affiliations:** 1 Department of Physical Education and Sport Sciences, Faculty of Humanities, Tarbiat Modares University, Tehran, Iran; 2 Department of Exercise Physiology, Faculty of Sport Sciences, University of Guilan, Rasht, Iran; 3 Department of Social Welfare, Institute for Humanities and Cultural Studies, Tabatabaei University, Tehran, Iran; 4 Department of Cancer Quality of Life, Breast Cancer Research Center, Motamed Cancer Institute, ACECR, Tehran, Iran

**Keywords:** Breast cancer, Home-based exercise, Quality of life

## Abstract

**Background:**

Breast cancer patients are recommended to engage in regular exercise. In developing countries, where there is a lack of facilities to offer specialized, supervised exercise for this population, regularly exercising might be a challenge. We aimed to evaluate the effectiveness of a home-based intervention in this population.

**Methods:**

Breast cancer survivors were randomly assigned to either the home-based exercise program or the usual care group. Exercise intervention included walking, balance, and stretch exercises, along with weekly follow-up telephone calls. Quality of life (QOL) was evaluated using EORTC QLQ-C30 and EORTC QLQ-BR23 questionnaires and the predicted VO_2_ peak was measured using the Ebbeling submaximal treadmill test.

**Results:**

Eighty-nine patients were enrolled in the study. Reported minutes of exercise gradually increased from 40.7 min per week in week 1 to 116.9 min per week in week 12. This intervention improved global QOL (*P* = 0.001), social functioning (*P* = 0.04), and the predicted VO_2_ peak (*P* = 0.01).

**Conclusion:**

This home-based exercise regime effectively increased quality of life and physical activity levels.

**Trial registry:**

Iranian Registry of Clinical Trials identifier: IRCT20140810018746N1, prospectively registered 08/01/2018, https://en.irct.ir/trial/27959.

**Supplementary Information:**

The online version contains supplementary material available at 10.1186/s13102-023-00710-7.

## Background

Breast cancer is the most frequently diagnosed cancer and the leading cause of cancer-related death among women worldwide and in the Islamic Republic of Iran [1]. However, advances in early diagnosis and treatment have increased survival rates. This means that a greater number of survivors are now facing the debilitating effects of anticancer therapies, which have been shown to significantly impact the quality of life (QOL) of survivors. Health-related quality of life (HRQOL) is compromised during breast cancer, lasting for years after the completion of treatments [2, 3]. If not addressed, compromised HRQOL can impact various QOL domains, potentially putting survivors at a greater risk for the development of other comorbidities, including cardiovascular disease, diabetes, and even secondary cancer [4–6].

Exercise has been shown to offer a multitude of benefits to breast cancer patients across the disease continuum, including during treatment [7, 8] and after the completion of major treatments and beyond [9, 10]. Especially, there is strong evidence for the effectiveness of exercise in improving QOL and physical functioning [11].

Onsite supervised training, as opposed to home-based, may be more effective and therefore the recommended setting for benefiting from the health impacts of physical activity but might not necessarily be the most preferred one among cancer survivors [12]. Moreover, facilities specializing in providing supervised exercise training for cancer survivors are lacking in Iran, although research indicates a lack of tendency among breast cancer survivors to attend public facilities even if such facilities were available to them [13, 14]. Besides, the social, economic, and cultural status of countries have a huge impact on the physical activity level of people as evident from the higher level of inactivity in developing countries compared with European countries [15]. Still, other factors might contribute to a sedentary lifestyle, or disrupt an already active one, as was the case with the recent emergence of the COVID-19 pandemic, which put the world in a state of lockdown for almost 2 years—and the possibility of the emergence of other pandemics is not ruled out.

A good deal of research has looked into the effectiveness of homebased exercise in improving various components of physical fitness and quality of life in breast cancer survivors—a recent meta-analysis included 13 randomized controlled trials of such interventions [16]. However, most of these studies have been conducted in Europe and North America, while breast cancer survivors constitute an understudied population in Iran. The purpose of this study, then, was to examine the effectiveness of a 12-week home-based exercise program in improving QOL and components of physical fitness in previously sedentary breast cancer survivors. Moreover, we aimed to examine the potential variables which might affect the adherence of participants to that trial.

## Methods

### Design

This was a 2-arm (parallel with an allocation ratio of 1:1) randomized controlled clinical trial that was conducted at Motamed Breast Cancer Institute, Academic Center for Education, Culture and Research, Tehran, Iran. The study was conducted in agreement with the Declaration of Helsinki and was approved by the Ethics Committee of Breast Cancer Research Center. All participants provided written informed consent after being completely briefed about the purpose of the study.

### Participants

Female breast cancer patients being treated at Motamed breast cancer clinics were recruited between 2018 and 2020. Patients were eligible if they had stage I, II, or IIIA breast cancer, were at most one year after treatment (e.g., surgery, chemotherapy, or radiation therapy), were between 19 and 59 years of age, had a body mass index (BMI) between 18 and 30 kg/m^2^, were not engaged in any type of physical activity (as determined via taking a thorough history of physical activity), and had no cardiovascular, neurologic, orthopedic, or metabolic conditions contraindicating physical activity.

The sample size was estimated at 84, with the assumption of a 20% dropout during the trial, an α level of 0.05, and a power of 80%. Consulting the literature, the standard deviation (SD) and a statistically significant increase in QOL scores were assumed at 27.7 and 20, respectively [6, 17].

The participants were randomly allocated to one of the concealed packets provided for block randomization. In each block of size 2, the patients were randomly allocated to a home-based exercise group or a usual care control group. In a briefing session, two exercise physiologists taught them how to perform the exercise program and record their training sessions at home. Quality of life, exercise capacity, and anthropometric indices were measured at baseline and the end of the intervention.

### Exercise intervention

The exercise package was developed based on an expert focus group discussion held by the same research team [18], incorporating walking, balance exercises, and stretches to best cater for the needs of breast cancer survivors (Supplement Table 1). The intervention group began the exercise regime at a volume of 15 min per session, 2 days per week, and gradually increased the duration to reach the target volume of 50–60 min per session, at least 3 days per week, or 30–40 min per session, 5 days per week, for a total period of 3 months. They were instructed to monitor their exertion intensity using the talk test (being able to converse comfortably during exercise) to maintain a moderate intensity of the exertion. Participants were asked to enter the details of their workout in a training logbook.

A weekly telephone call was made to track the subjects’ adherence to exercise and enquire about potential issues or questions they faced during exercise. In case of any question or exercise-related problem, a telephone appointment with exercise experts would be set to address the issues. The control group was checked on through the phone once a month.

### Outcome measures

The main outcome of the research was QOL, which was measured using the European Organization for Research and Treatment of Cancer (EORTC) QLQ-C30 and EORTC QLQ-BR23. These two questionnaires have been translated into Farsi [6]. The EORTC QLQ-C30 and QLQ-BR23 assess cancer-specific QOL and breast cancer–specific QOL, respectively [19].

Adherence rate of participants was assessed by calculating the total weekly minutes of exercise using the exercise logs.

Exercise capacity was assessed using a single-stage submaximal treadmill walking test [20]. Before the test, patients filled out the PAR-Q questionnaire [21]. Resting heart rate and blood pressure were obtained after a 2-min rest. Participants wore a heart rate monitor (Polar, USA, Lake Success, New York) and sat for two minutes before resting heart rate and blood pressure measures were obtained. The test started with a 4 min warm-up on a treadmill at 2.0 to 4.5 km/h based on patients’ comfort. After the warm-up stage, the participants walked on the treadmill at a 5% incline for 4 min. The heart rate and the walking speed during the last minute were recorded and placed in the formula to estimate VO_2_ peak.

Body mass and height were measured to calculate BMI. Hip and waist circumferences were measured to calculate the waist to hip ratio. Mean arterial pressure was estimated using the formula [(2 × diastolic blood pressure) + systolic blood pressure] / 3. Adherence to exercise was calculated based on minutes of walking and exercising reported by patients. All measurements were done at Breast Cancer Research Center, Motamed Cancer Institute.

### Statistical analyses

Data were analyzed with SPSS version 22 (SPSS Inc., Chicago, IL). To compare the two groups, we used Pearson’s chi-square for categorical variables and the independent-samples *t* test for quantitative variables. Since the QOL scores were not distributed normally, baseline scores were compared with the Mann-Whitney U test, and the mean changes from baseline to week 12 were compared using the Kruskal-Wallis test.

Logistic regression modeling was used to study the interaction effect of possible predictors of exercise adherence. According to the experts’ opinion, probable effective variables consisting of age, education, marital status, occupation, BMI, waist to hip, weight, estimated VO_2_ max, hormone therapy, and QOL at baseline entered in the model. The Hosmer-Lemeshow test was used to test the goodness of fit for our model.

## Results

**Fig. 1:**
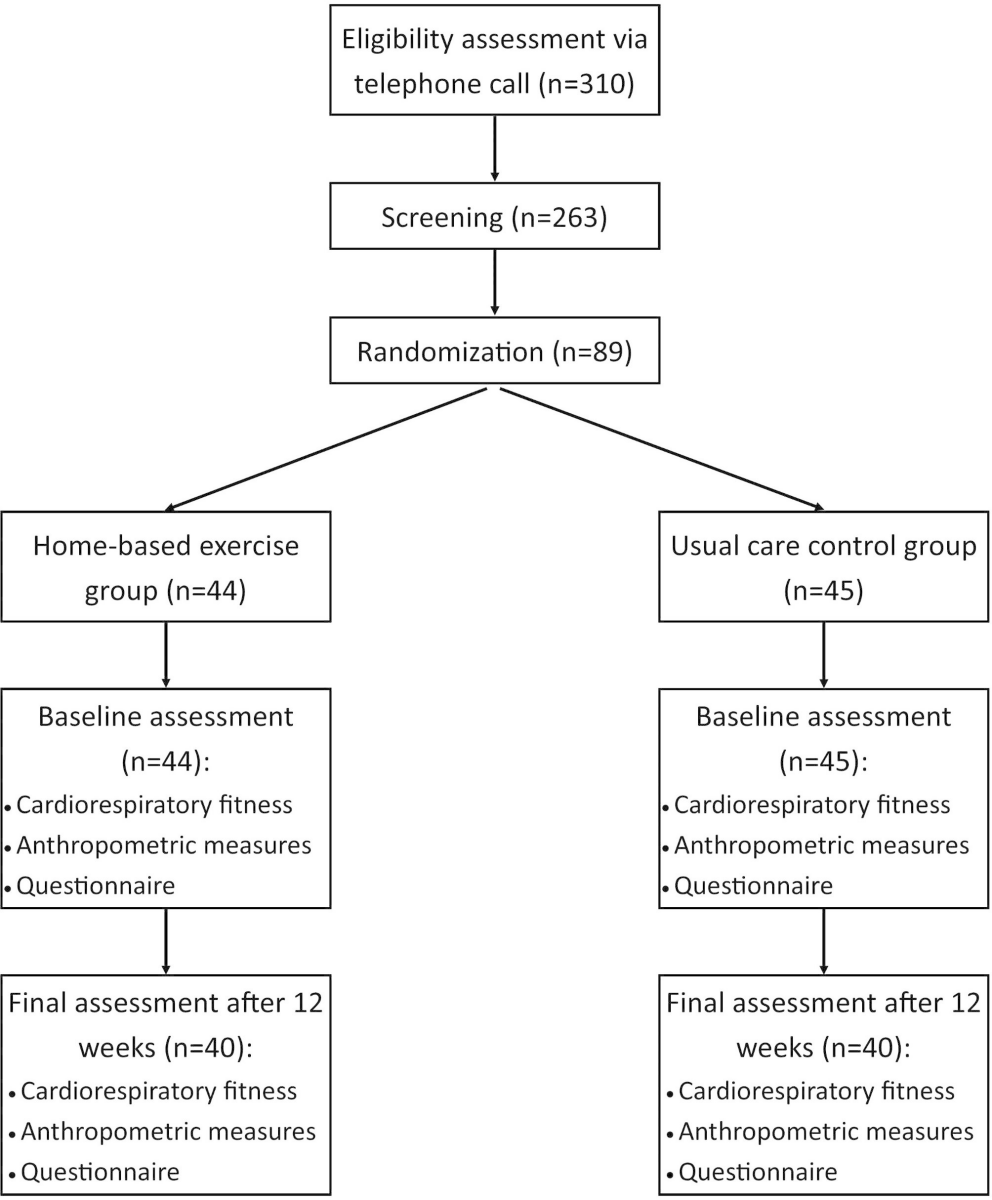
Flow of the participants

The flow of participants is presented in Fig. 1. The overall retention rate for our study was 89%, and it did not significantly differ between the exercise (90%) and the control group (88%). The reasons to drop out of the study were losing the interest to keep on (n = 4), family issues (n = 2), moving to other cities (n = 2), and occurrence of metastasis (n = 1).

Table 1 presents the baseline characteristics of the participants. Briefly, they were middle-aged, mostly post-menopausal, overweight, with moderate tumor load. There were no significant baseline differences between the exercise and control groups in demographic, physiologic, or medical characteristics.

**Table 1:** Demographic, physiologic, and medical characteristics of participants

	Overall (n = 89 )	Exercise group (n = 44 )	Control group (n = 45 )	*P* value
Age^*^	45.8 (8.2)	45.05 (7.5)	46.6 (8.8)	0.21
Not married, n (%)	14 (15.7%)	5 (11.4%)	9 (20%)	0.36
Graduated, n (%)	22 (24.7%)	12 (27.3%)	10 (22.2%)	0.68
Employed, n (%)	13 (14.6%)	6 (13.6%)	7 (15.6%)	0.47
Having no children, n (%)	17 (19.1%)	6 (6.2%)	11 (12.4%)	0.44
Postmenopausal, n (%)	54 (60.7%)	30 (68.2%)	24 (53.3%)	0.11
Body mass index, kg/m^2^	28.5 (4.9)	27.7 (5.2)	29.4 (4.4)	0.97
Waist to hip ratio	0.9 (0.1)	0.9 (0.2)	0.8 (0.1)	0.57
Estimated VO_2_ max, ml∙kg^-1^∙min^-1^	22.4 (4.4)	21.9 (4.3)	22.9 (5.5)	0.33
Systolic blood pressure, mm Hg	12.08 (1.7)	11.6 (1.5)	12.5 (1.7)	0.24
Diastolic blood pressure, mm Hg	7.9 (1.1)	7.6 (1.07)	8.3 (1.2)	0.51
Surgery type, n (%)				
Modified radical mastectomy	29 (32.6%)	10 (22.7%)	19 (42.2%)	0.50
Breast-conserving surgery	60 (67.4%)	34 (77.3%)	26 (57.8%)	
Stage, n (%)				
I	20 (22.5%)	11 (25%)	9 (20%)	0.44
II	56 (62.9%)	29 (65.9%)	27 (60%)	
III	3 (3.4%)	1 (2.3%)	2 (4.4%)	
Current hormone therapy, n (%)	62 (70.5%)	29 (65.9%)	33 (75%)	0.24
Current Herceptin therapy, n (%)	18 (20.5%)	4 (9.1%)	14 (31.8%)	**0.08**

^*^ Data are presented as mean (SD), unless otherwise stated

Details of exercise adherence over the 12 weeks are presented in Table 2. The mean reported time at exercise gradually increased from 40.7 ± 24.40 min in week 1 to 116.9 ± 89.33 min in week 12. Although the goal was set on 150 min per week in week 12 and only 42.5% of it was achieved, the increase in physical activity levels from week 1 to week 12 was statistically significant (*P* < 0.001). Adherence rate was high during weeks 1 to 6, but from week 7, about 50% of the participants were not able to keep up with the planned weekly goal.

**Table 2:** Details of exercise adherence in the intervention group during the trial

	Weekly minutes at exercise, mean (SD)	Goal, min	Percent meeting the goal
Week 1	40.75 (24.40)	20	85
Week 2	46.87 (23.63)	30	77.5
Week 3	58.25 (29.40)	30	87.5
Week 4	74.75 (34.52)	40	82.5
Week 5	76.12 (43.12)	60	70
Week 6	90.25 (54.95)	60	72.5
Week 7	93.50 (57.42)	100	52.5
Week 8	105.12 (65.73)	100	52.5
Week 9	97.00 (74.05)	125	35
Week 10	97.37 (72.29)	125	35
Week 11	115.25 (80.16)	150	37.5
Week 12	116.87 (89.33)	150	42.5

Table 3 lists the physiological changes over the 12 weeks. Predicted VO_2_ peak increased in the exercise group compared with the control group (*P* = 0.01). Other physiological measures did not change between groups across time.

**Table 3:** Physiological changes over the 12 weeks

	Exercise group (n = 40)		Control group (n = 40)		*P* value
	Baseline	After 12 weeks	Baseline	After 12 weeks	
Predicted VO_2_ max	19.4 (0.5)	20.9 (0.6)	19.4 (0.5)	19.4 (0.6)	**0.01**
Body mass index	27.7 (0.8)	27.4 (0.8)	29.4 (0.7)	29.3 (4.4)	0.11
Weight	70.2 (2.09)	69.5 (2.04)	74.1 (0.6)	74.1 (1.6)	0.18
Weight to hip ratio	0.9 (0.03)	0.8 (0.01)	0.8 (0.01)	0.8 (0.01)	0.33
Mean arterial pressure	89.7 (11.4)	89.7 (8.3)	97 (13.11)	94 (13.25)	0.15

Data are presented as mean (SE)

Global QOL score increased by 25% and 7.4% in the intervention and the control group, respectively (*P* = 0.001) (Table 4). Also, a significant improvement was observed for the exercise group in social functioning (*P* = 0.04). Other subscales, including physical functioning, did not differ significantly between the two groups. No adverse effects were reported during the intervention. Looking at what would predict adherence to exercise, we did not find any significant predictor in the model.

**Table 4:** Effect of exercise training on quality of life outcomes

	Exercise group (n = 40)		Control group (n = 40)		*P* value
Variable	Baseline	After 12 weeks	Baseline	After 12 weeks	
**EORTC QLQ-C30**					
Global QOL	50 (31.2)	75 (25)	66.6 (33.3)	75 (16)	**0.001**
**Functional scales**					
Physical functioning	76.6 (20)	86.6 (18.3)	86.6 (20)	86.6 (18.3)	0.06
Role functioning	83.3 (33.3)	100 (18.7)	100 (33.3)	100 (29.1)	0.85
Emotional functioning	66.6 (25)	83.3 (39.5)	66.6 (41.6)	75 (25)	0.60
Cognitive functioning	83.3 (33.3)	83.3 (33.3)	83.3 (33.3)	83.3 (16.6)	0.28
Social functioning	83.3 (33.3)	100 (33.3)	100 (33.3)	83.3 (33.3)	**0.04**
**Symptom scales**					
Fatigue	33.3 (33.3)	27.7 (30.5)	22.2 (41.6)	11.1 (30.5)	0.76
Nausea and vomiting	0 (0)	0 (0)	0 (0)	0 (0)	0.33
Pain	33.3 (33.3)	16.6 (33.3)	16.6 (33.3)	16.6 (16.6)	0.35
Dyspnea	0 (33.3)	0 (33.3)	0 (33.3)	0 (33.3)	0.50
Insomnia	33.3 (33.3)	0 (33.3)	16.6 (33.3)	0 (33.3)	0.30
Appetite loss	0 (0)	0 (0)	0 (33.3)	0 (25)	0.69
Constipation	0 (33.3)	0 (33.3)	0 (33.3)	0 (25)	0.63
Diarrhea	0 (0)	0 (0)	0 (0)	0 (0)	0.75
Financial difficulties	33.3 (66.6)	33.3 (66.6)	33.3 (33.3)	33.3 (33.3)	0.22
**EORTC QLQ-BR23**					
**Functional scales**					
Body image	91.6 (39.5)	91.6 (33.3)	91.6 (33.3)	91.6 (25)	0.78
Sexual functioning	66.6 (33.3)	66.6 (33.3)	66.6 (29.1)	66.6 (33.3)	0.72
Sexual enjoyment	66.6 (33.3)	66.6 (33.3)	66.6 (33.3)	66.6 (33.3)	0.73
Future perspective	33.3 (58.3)	66.6 (33.3)	66.6 (33.3)	66.6 (58.3)	0.74
**Symptom scales**					
Systemic therapy side effects	21.4 (17.8)	19 (14.2)	19 (28.5 )	14.2 (16.6)	0.52
Breast symptoms	16.6 (16.6)	8.3 (22.9)	16.6 (31.2)	8.3 (25)	0.71
Arm symptoms	16.6 (22.2)	22.2 (30.5)	22.2 (22.2)	22.2 (22.2)	0.39
Upset by hair loss	0 (33.3)	0 (0)	0 (33.3)	0 (33.3)	0.91

Data are presented as median (IQR)

Abbreviation: QOL, quality of life

## Discussion

This study examined the effectiveness of a 12-week home-based exercise regime in breast cancer patients. The mean reported time at exercise gradually increased from 40.7 min in week 1 to 116.9 min in week 12, and the increase was significant.

QOL improved in the exercise group after 3 months of training. QOL is an important aspect of breast cancer care as it is mainly considered a prognostic factor [5]. QOL measured by the EORTC questionnaire consisted of several components. Social functioning improved in the exercise group, while it declined in the control group. Social well-being is considered an important prognostic factor during the first year after cancer diagnosis [24]. Interestingly, although this exercise program was home-based and the participants exercise in home isolation, social functioning significantly improved in the exercise group. Exploring possible factors contributing to this phenomenon could be a subject of future studies. Despite our hypothesis, physical functioning did not change significantly over the trial period, although the exercise group experienced a 13% increase in physical functioning. Collectively, based on the results of this study, the home-based exercise program was effective in increasing global QOL and social functioning in breast cancer survivors.

The recommended, safest approach for starting a physical activity program is “start low and go slow” [22]. But how slow should it be? We found that although the minutes of exercise increased gradually, the adherence to the goal we set declined gradually. We expected, based on a previous study by Pinto et al. [23], that sedentary breast cancer survivors could start with 20 min and gradually increase to 150 min by week 11. It is possible that the goal we set for these participants was not appropriate for them and that we might need to reconsider our goal set for an exercise program in breast cancer patients.

Breast cancer treatments lead to a drastic decrease in cardiorespiratory fitness [25], which might remain for years after the completion of treatment [26]. Supervised facility-based exercise interventions have been reported to bring about significant increases in VO_2_ peak [27, 28]. Our home-based exercise program was also able to elicit beneficial effects on predicted VO_2_ peak, an indirect measure of cardiorespiratory fitness. However, the improvement was not as high as that observed in previous studies. The reason for this is that the mode of exercises in our study is, by nature, not expected to bring about great improvement in cardiorespiratory fitness.

This study has limitations and strengths. It lacked an objective monitoring of adherence to exercise. Home-based interventions with close and objective monitoring of adherence will contribute to the precise prescription of home-based exercise programs for breast cancer survivors. Further, for the assessment of cardiorespiratory fitness, we used the estimated VO_2_ test, which might not be considered a precise test compared with direct methods. Another limitation, which may have implications for adherence, was that the exercise prescription was the same for all the patients, while a tailored prescription in terms of intensity and volume may have had a greater chance of being adhered to. Also, it would have been more insightful if the focus group that informed the development of exercise program had included breast cancer survivors. Since those with lived experience of breast cancer might have a unique and extremely important insight into the disease, this could have resulted in the development of a more feasible protocol for home-based exercise. On the strengths side, we could mention the randomized controlled trial design of the study, the low attrition rate, and the relatively large sample size. Moreover, this study was conducted in an underrepresented group, i.e., breast cancer survivors in a Middle Eastern country, and the results could help with the designing of interventions to increase HRQOL in this population.

## Conclusion

The home-based exercise package developed in this study was associated with beneficial effects on global QOL, social functioning, and cardiorespiratory health of breast cancer survivors. This exercise package can be prescribed, with some adjustments for each individual, to increase the physical activity levels of breast cancer patients to help maintain some components of physical fitness in the absence of specialized fitness centers for this population.

### Electronic supplementary material

Below is the link to the electronic supplementary material.

Supplementary Material 1

## Data Availability

The study data are available from the corresponding author upon reasonable request.

## Abbreviations

QOL: Quality of life
HRQOL: Health-related quality of life
BMI: Body mass index
EORTC: European Organization for Research and Treatment of Cancer
