# Supplementary material for: Effectiveness of home-based exercise in breast cancer survivors: a randomized clinical trial
Source: BMC Sports Sci Med Rehabil. 2023 Aug 7;15:96. doi: 10.1186/s13102-023-00710-7 (PMC10405487; doi:10.1186/s13102-023-00710-7)
Supplement: Supplementary file 1 — Supplementary Material 1 [file 13102_2023_710_MOESM1_ESM.docx]

Supplement 1. Exercises included in the exercise package

| Warm-up | |
| --- | --- |
| 1 | Shoulder pendulum |
| 2 | Shoulder circles |
| 3 | Scapula squeezes |
| 4 | Pelvic tilt |
| Balance | |
| 1 | Heel raises |
| 2 | Walk a narrow path |
| 3 | Braiding |
| 4 | Single-leg stance |
| Stretches-upper body | |
| 1 | Single-arm overhead stretch |
| 2 | Wall climb: front |
| 3 | Wall climb: side |
| 4 | Butterfly stretch |
| 5 | Seated side stretch |
| 6 | Single-arm wall stretch |
| 7 | Overhead clasped hands stretch |
| 8 | Chest stretch |
| 9 | Shoulder stretch |
| Stretches-core | |
| 1 | Supine stretch |
| 2 | Full-body stretch |
| 3 | Cat-camel |
| 4 | Supine torso rotation |
| Stretches-lower body | |
| 1 | Kneeling hip flexor stretch |
| 2 | Supine hip flexor stretch |
| 3 | Supine hamstring stretch |
